# Role of Hepatic Progenitor Cells in Nonalcoholic Fatty Liver Disease Development: Cellular Cross-Talks and Molecular Networks

**DOI:** 10.3390/ijms141020112

**Published:** 2013-10-09

**Authors:** Guido Carpino, Anastasia Renzi, Paolo Onori, Eugenio Gaudio

**Affiliations:** 1 Department of Anatomical, Histological, Forensic Medicine and Orthopedics Sciences, Sapienza University of Rome, Rome 00161, Italy; E-Mails: anastasia.renzi@uniroma1.it (A.R.); paolo.onori@uniroma1.it (P.O.); eugenio.gaudio@uniroma1.it (E.G.); 2 Department of Movement, Human and Health Sciences, University of Rome “Foro Italico”, Piazza Lauro De Bosis 6, Rome 00135, Italy

**Keywords:** nonalcoholic fatty liver disease, hepatic progenitor cell, hepatic stellate cells, macrophages, kupffer cells, fibrogenesis

## Abstract

Nonalcoholic fatty liver disease (NAFLD) includes a spectrum of diseases ranging from simple fatty liver to nonalcoholic steatohepatitis, (NASH) which may progress to cirrhosis and hepatocellular carcinoma. NASH has been independently correlated with atherosclerosis progression and cardiovascular risk. NASH development is characterized by intricate interactions between resident and recruited cells that enable liver damage progression. The increasing general agreement is that the cross-talk between hepatocytes, hepatic stellate cells (HSCs) and macrophages in NAFLD has a main role in the derangement of lipid homeostasis, insulin resistance, danger recognition, immune tolerance response and fibrogenesis. Moreover, several evidences have suggested that hepatic stem/progenitor cell (HPCs) activation is a component of the adaptive response of the liver to oxidative stress in NAFLD. HPC activation determines the appearance of a ductular reaction. In NASH, ductular reaction is independently correlated with progressive portal fibrosis raising the possibility of a periportal fibrogenetic pathway for fibrogenesis that is parallel to the deposition of subsinusoidal collagen in zone 3 by HSCs. Recent evidences indicated that adipokines, a class of circulating factors, have a key role in the cross-talk among HSCs, HPCs and liver macrophages. This review will be focused on cellular cross-talk and the relative molecular networks which are at the base of NASH progression and fibrosis.

## 1. Introduction

Nonalcoholic fatty liver disease (NAFLD) is an increasingly recognized condition that includes a wide spectrum of diseases ranging from simple fatty liver to nonalcoholic steatohepatitis (NASH), and which may progress to end-stage liver disease (cirrhosis) and hepatocellular carcinoma. The pathological characteristic resembles that of alcohol-induced liver injury, but it occurs in patients who do not abuse alcohol. NAFLD is characterized by hepatic accumulation of triglycerides (*i.e.*, steatosis), in combination with hepatic inflammation (NASH) [1]. Nonalcoholic fatty liver disease affects 10%–24% of the general population in Western world. The prevalence increases to 57.5%–74% in obese persons [1]. Nonalcoholic fatty liver disease affects 2.6% of children and 22.5%–52.8% of obese children [2,3]. NAFLD has been considered as the hepatic manifestation of the metabolic syndrome (MS) [4,5].

The mechanisms underlying NASH development have been poorly characterized. Recent evidences suggest that NASH progression is due to several interactions between resident and recruited cells. The aims of the present review are to discuss the recent mechanisms at the base of cross-talk among injured hepatocytes, hepatic progenitor cell (HPC), hepatic stellate cells (HSCs), and macrophages in NAFLD and their role in NASH development and fibrogenesis.

## 2. Histo-Pathological Aspects of NAFLD

The diagnosis of nonalcoholic steatohepatitis (NASH) is established by the presence of a characteristic pattern of steatosis, inflammation, and hepatocellular ballooning on liver biopsies in the absence of significant alcohol consumption [6]. The value of establishing a diagnosis of NASH is to identify individuals who are at risk for progressive liver disease to the point of cirrhosis and death from chronic liver disease. For this reason, a scoring system for nonalcoholic fatty liver disease (NAFLD) was developed and validated by the National Institute of Diabetes and Digestive and Kidney Diseases (NIDDK) which sponsored Nonalcoholic Steatohepatitis Clinical Research Network (NASH CRN) Pathology Committee [7]. The proposed methodology for the histological scoring include the division of lesions of active and potentially reversible injury (“grade”) in the NAFLD Activity Score (NAS) and those potentially less reversible and characterized by collagen deposition and architectural alterations that may evolve toward more permanent parenchymal remodeling (“stage”). The proposed NAS also clearly separates the three lesions that comprise grade: steatosis, lobular inflammation, and ballooning. The histological features were grouped into five broad categories: steatosis, inflammation, hepatocellular injury, fibrosis, and miscellaneous features. The Pathology Committee suggested classification of NAFLD into the following types: type 1, simple steatosis; type 2, steatosis and inflammation; type 3, steatosis and cell swelling (ballooning); type 4, steatosis, cell swelling (ballooning), and fibrosis. Progression to cirrhosis is found predominantly in types 3 and 4, both of which correspond to the typical histopathological picture of NASH [8–10]. Traditionally, simple steatosis has been considered a relatively benign lesion, while patients with steatohepatitis have a high risk to progress toward advanced fibrosis or cirrhosis and are at increased risk of death [11].

Moreover, in recent publication from the NASH CRN, the diagnosis of definite SH or the absence of SH based on evaluation of patterns as well as individual lesions on liver biopsies does not always correlate with threshold values of the semiquantitative NAS [6]. In this light, the NAS score cannot be used as a replacement for the diagnosis of NASH for clinical purposes. Accordingly, a microscopic diagnosis based on overall pattern of injury (steatosis, hepatocyte ballooning, inflammation) as well as the presence of additional lesions (such as zonality of lesions, portal inflammation, and fibrosis) should be assigned to each case [6]. The assignment of a diagnostic category should be based on the consensus recognition of the distinctive features of steatohepatitis, independent of the degree of NAFLD severity indicated by the NAS. In this way, biopsies can be subdivided into the following categories: not steatohepatitis (not-SH), definite steatohepatitis (definite-SH) or borderline SH [6].

## 3. Liver Fibrosis in NAFLD and Hepatic Stellate Cell Activation

Liver fibrosis represents the final common pathway of almost all types of chronic liver diseases. Activated hepatic stellate cells (HSCs) and hepatic myofibroblasts (MFs) are the key cells implicated in the accumulation of extracellular matrix materials, including type I collagen [12].

**Figure 1:**
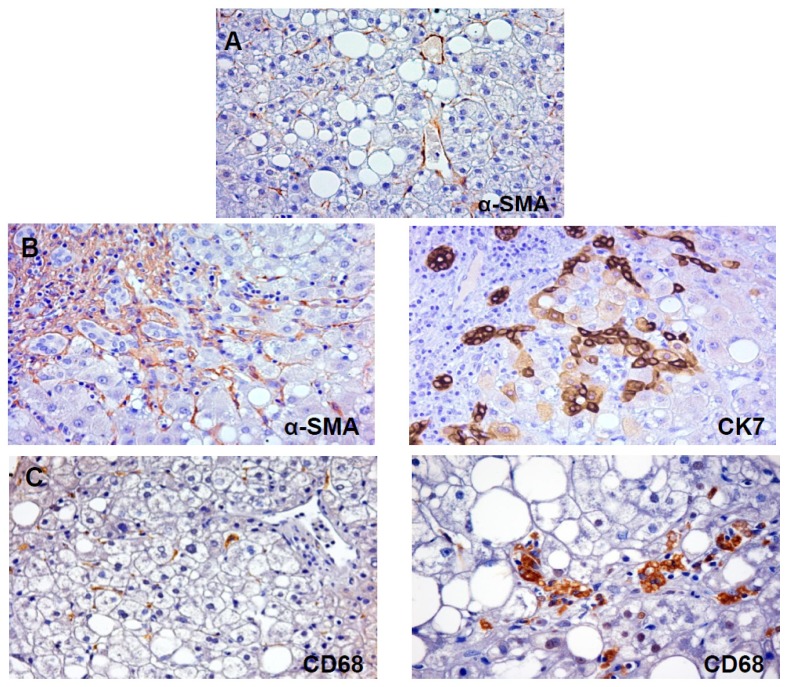
(**A**) Immunohistochemistry for α-smooth muscle actin (α-SMA) in non-alcoholic fatty liver disease (NAFLD). In NAFLD, pericentral fibrogenesis is due to activation of hepatic stellate cells (HSCs) which acquire α-SMA positivity. Original Magnification: 20×; (**B**) Immunohistochemistry for α-smooth muscle actin (α-SMA) and Cytokeratin (CK)-7 in NAFLD. In advanced stages of NAFLD, periportal fibrogenesis is present. In this case, α-SMA positive myofibroblasts surround CK7+ reactive ductules at the periphery of portal spaces. Original Magnification: 20×; (**C**) Immunohistochemistry for CD68 in NAFLD. CD68 is specifically expressed by Kupffer cells and macrophages which are distributed throughout entire liver lobule both at pericentral and periportal position. Macrophage foam cells are clearly recognized in left image. Original Magnification: 20× (**right**) and 40× (**left**).

Hepatic stellate cells are located in the sub-endothelial (Disse’s) space, between the hepatocytes and the anti-luminal side of sinusoidal endothelial cells. HSCs comprise approximately one-third of the non-parenchymal cell population and almost 15% of the total number of resident cells in normal liver [13]. In a healthy liver, HSCs are quiescent cells and contain numerous vitamin A lipid droplets, constituting the largest reservoir of vitamin A in the body [14]. When the liver is injured due to viral infection or hepatic toxins, HSCs receive signals secreted by damaged hepatocytes and immune cells, causing them to trans-differentiate into activated myofibroblast-like cells [13,15,16]. Stellate cell “activation” refers to the conversion of a resting vitamin A-rich cell to one that is proliferating, fibrogenic, and contractile (expression of α-smooth-muscle actin: Figure 1) [16,17]. Though it is known that mesenchymal cell populations contribute to extracellular matrix accumulation, stellate cell activation remains the most dominant pathway leading to hepatic fibrosis. Activated Kupffer cells, infiltrating monocytes, activated and aggregated platelets, and damaged hepatocytes are the sources of platelet-derived growth factor and transforming growth factor (TGF)-β1, which trigger the initiation of intracellular signaling cascades that lead to the activation of HSCs [18]. The quiescent HSCs may develop adipogenic or myogenic characteristic during the trans-differentiation process [19]. The different directions of trans-differentiaton are determined by the imbalance between clusters of adipogenic genes and myogenic genes. The expression of adipogenic genes is down-regulated under the stimulus of ischemia and inflammation. Peroxisome proliferator-activated receptor gamma (PPAR-γ) is the principal adipogenetic gene. On the other hand, activated HSCs express myogenic genes acquiring a myofibroblast-like phenotype and start to actively secrete extracellular matrix (ECM) components, including fibrillar collagens (collagen I and III) [20]. Moreover, HSCs are the main source of tissue inhibitors of metalloproteinases (TIMPs), which may decreases ECM degradation through suppression of the matrix metalloproteinases (MMPs) activities. Besides HSCs, it has been demonstrated that hepatocytes are also a source of TIMPs and other matrix modulators and, therefore, they could have a role in processes of fibrogenesis and fibrosis regression [21]. In general, the altered balance between ECM synthesis and degradation leads fibrogenesis [22].

Although key pathways of HSCs activation are common to all forms of liver injury and fibrosis, disease-specific pathways also exist. In addition to the transforming growth factor (TGF-β1) signaling pathway, which is known to play major role in the activation of HSCs in liver fibrosis, many other pathways are implicated in liver fibrosis in NAFLD, such as the Hedgehog (Hh) [23,24], PI3K/AKT, and JAK/STAT/ERK signaling pathways [18]. Moreover, extracellular molecules, such as lypopolysaccharide (LPS), tumor necrosis factor (TNF)-α, interleukin (IL)-1β, and reactive oxygen species (ROS), can activate fibrogenic gene expression [12,18]. Leptin binds its receptor, activating the JAK2/STAT3 pathway and inducing matrix deposition through increased expression of TIMPs. Leptin also inhibits matrix degradation through decreased expression of MMPs [25]. Adiponectin derived from adipose tissue suppresses the proliferation and migration of HSCs [25]. Finally, TLR4 has a key role in activating HSCs through a NF-κB-dependent pathway [26,27].

Although the role of HSCs activation in NAFLD has not been completely clarified, several studies have reported increased HSCs activation in NASH [28]. The well-known role of HSCs in the pathogenesis of liver fibrosis suggests that they may play a key role in NASH-related hepatic fibrosis, in which ECM deposition in the pericellular space forms a characteristic “chicken-wire” pattern [23,29].

**Figure 2:**
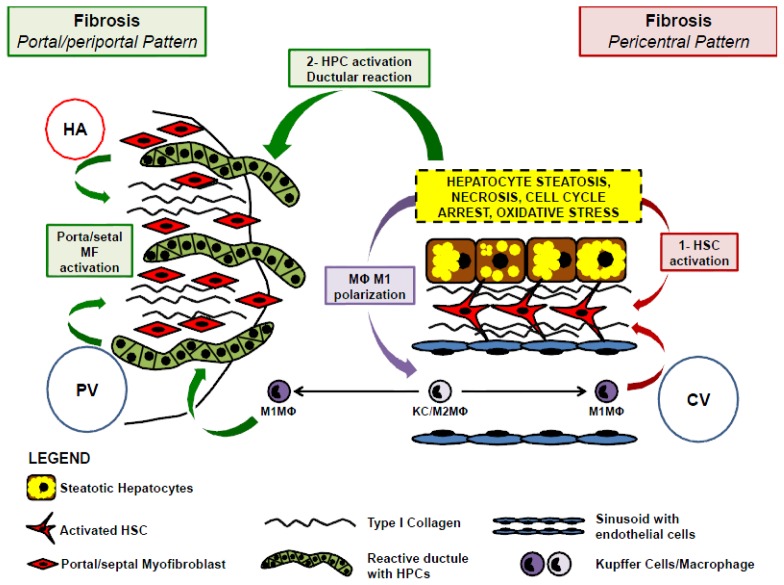
Cartoon indicating possible cellular cross-talks among hepatic stellate cells (HSCs), hepatic progenitor cells (HPCs) and Kupffer cells/macrophage (KC/MΦ) in NAFLD progression. Two distinct fibrogenic pathways are present in NAFLD. Pericentral fibrogenesis is due to activation of HSCs by damaged hepatocytes. On the other hand, hepatocyte damaging could stimulate HPC proliferation, thus resulting in the appearance of ductular reaction (DR); in turn, DR activates portal myofibroblasts (MF) which are responsible of periportal fibrogenesis. Finally, KC/MΦ polarization toward M1 phenotype could be involved in both pathways since M1-MΦs are able to stimulate HSCs and HPCs. PV = portal vein; CV = central vein; HA = hepatic artery.

In NAFLD, two different patterns (centrilobular and portal) of fibrosis have been individuated (Figure 2). In adults, a centrilobular pattern of subsinusoidal fibrosis is typical [8,30]. The mechanism proposed for triggering fibrogenesis in NASH is lipotoxicity [31]. Hepatocellular damage results in the induction of pro-inflammatory and profibrogenic cytokines [32,33], activation of adjacent HSCs and subsequent deposition of type I collagen. In NASH, this typically occurs within the lobules at the site of hepatocellular injury, resulting in a pericellular, subsinusoidal fibrosis maximal in centrilobular areas [8].

Paediatric disease, on the other hand, is often characterized by pure portal fibrosis and may be accompanied by a predominant periportal steatosis and portal inflammation [30,34,35]. A predominant portal fibrosis occasionally occurs in adults as well [6,36]. Moreover, progression of fibrosis in adult NASH is characterized by portal fibrosis and periportal fibrous septa. In adult and paediatric NAFLD, therefore, portal fibrosis develops despite the lobular location of hepatocellular injury [6,36].

Heterogeneity of fibrosis patterns in non-alcoholic fatty liver disease supports the presence of multiple fibrogenic pathways. Chronic liver diseases are often characterized by activation of an alternative transit-amplifying compartment of periportal and bipotential hepatic progenitor cells (HPCs) that may be involved in the development of portal fibrosis pattern [37–39].

## 4. HPC Niches within Adult Intrahepatic Bile Duct Systems

In the liver, a resident stem cell compartment is present at the level of Canals of Hering which represent the smaller branches of intrahepatic biliary tree [40–42]. Hepatic stem/progenitor cells (HPCs or HpSCs, in humans) or oval cells (in rodents) are bipotential stem cells which are able to differentiate towards mature hepatocytes and cholangiocytes [40,43,44]. In adult human livers, hepatic progenitor cells are facultative stem cells with a low proliferating rate [45,46]. Even when the liver responds to injuries, the cell loss and mass is normally restored through the replication of hepatocytes and large cholangiocytes [47,48]. So, hepatic progenitor cells represent a reserve compartment that is activated only when the mature epithelial cells of the liver are continuously damaged or inhibited in their replication or in cases of severe cell loss. In these conditions, resident hepatic HPCs are activated and expand from the periportal to the pericentral zone giving rise to reactive ductules. Reactive ductules (or ductular reaction: DR) are strands of HPCs representing a trans-amplifying population with an highly variable phenotypical profile [39,40,43].

The role of HPCs to tissue turnover and regeneration is difficult to address in adult organs. Recently, several stem cell lineage-tracing tools have been developed to assess the location of HPCs and their involvement in liver regeneration [49,50].

In their paper, Furuyama and associates used *inducible* Cre technology under the control of the Sox9 transcriptional control elements and found Sox9+ HPCs in close proximity to the biliary tree in normal liver. Interestingly, when healthy animals were left for up to 12 months, the parenchyma of these animals was replaced by cells of a Sox9 origin, the putative HPCs, which are the predominant source of new hepatocytes in mouse liver homeostasis and afford near-complete turnover of the hepatocyte mass within six months [51]. They also showed that liver progenitor cells give rise to hepatocytes after two-thirds partial hepatectomy (2/3 PH) and carbon tetrachloride (CCl4) intoxication, both of which are experimental models believed to trigger hepatocyte regeneration only by self-duplication [52].

These findings are in controversy with the recent paper by Malato Y. *et al*. [53] and confuted by the study of Lamaigre and associates [54]. These lineage-tracing studies showed that newly formed hepatocytes derived from preexisting hepatocytes in the normal liver and that liver progenitor cells contributed minimally to hepatocyte regeneration after acute injury. This study supports the concept that liver progenitor cells contribute only minimally to normal hepatocyte turnover and to the regeneration of acutely lost hepatocytes. In this view, liver progenitor cells provide a backup system for injury states in which the proliferative capabilities of hepatocytes or cholangiocytes are impaired [55].

However, the paper by Furuyama [51] has the merit to definitely confirm the so-called “streaming liver hypothesis” demonstrating a streaming gradient of cells that arise at the portal tract and then divide and potentially migrate through the zones of the liver until they reach the central vein.

The culmination of these lineage-tracing strategies has resulted in an important recently published work by the Leclercq group [56]. Using osteopontin-1 as a marker, the authors demonstrate that HPCs express osteopontin (a glycoprotein that marks HPCs), emerge from bile duct, and are capable of directly differentiating into hepatocytes [56]. Importantly, HPCs regenerated hepatocytes following chronic hepatocyte injury but not following biliary injury, demonstrating that the microenvironment is critical for HPC expansion and fate choice.

## 5. HPC Microenvironment and Niche Modulation

**Figure 3:**
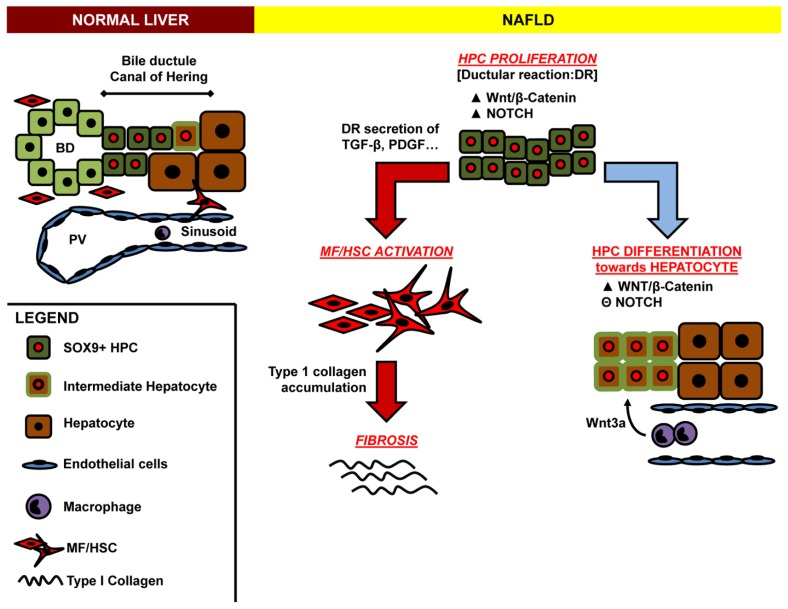
Cartoon indicating possible molecular cross-talks involving hepatic stellate cells (HSCs), hepatic progenitor cells (HPCs) and liver macrophages. In NAFLD, HPCs highly proliferate determining the appearance of ductular reaction (DR). HPC proliferation is determined by the up-regulation of both Wnt and Notch pathways. DR can produce several fibrogenetic factors such as TGF-β and PDGF which, in turn, activate portal myofibroblasts and HSCs to produce type 1 collagen. In parallel, the HPCs could differentiate towards hepatocytes; this process is characterized by a down-regulation of Notch signal and could be driven by macrophage Wnt3a secretion. PV = portal vein; BD = bile duct.

The local cellular microenvironment has a key role in achieving a defined progenitor specification and driving the acquirement of divergent cell fates in response to diverse diseases [49]. The study of well-described stem cell niches in other organs (intestinal, hair-follicle and the haematopoietic stem cell compartment) has indicated that Wnt and Notch signalling pathways are key regulators of stem cell proliferation and fate choice (Figure 3) [57].

In parallel, the activation of HPCs and the profile of the ductular reaction have been extensively investigated in developing liver, and in different human pathologies clarifying the role of signals involved in stem cell niche modulation. In human livers, the activation of the Wnt pathway plays a significant role in HPC expansion while the Notch pathway is involved in the fate choice of HPCs towards the cholangiocytic lineage [46,58].

During development, Notch signaling cascade is implicated in the formation of cholangiocytes and in the maturation and terminal patterning of the biliary tree [50,59]. Loss of Notch signaling in biliary development in mice, through genetic ablation of Jagged 1 (a Notch ligand) or haplo-sufficiency of Notch2, results in a reduction in biliary development and failure to pattern the biliary tree [50,60]. In parallel, the human congenital disease Alagille syndrome is characterized by a biliary paucity with failure to correctly resolve the ductal plate during development and is caused by mutations in Notch pathway components [50,60].

Wnt/β-catenin signaling in the developing liver plays critical roles in expansion of the liver bud and in formation of the definitive hepatoblasts, biliary proliferation, and hepatocyte maturation [50,61]. Interestingly, in the postnatal liver, activation of the canonical Wnt signaling pathway is required for the expansion of hepatocytes and is responsible for expansion of the liver [49,50].

Notch and Wnt are required for HPC differentiation, and their interaction is necessary for appropriate delineation of hepatocellular *versus* biliary fates [33]. In particular, during biliary regeneration, expression of Jagged 1 by myofibroblasts promoted Notch signaling in HPCs and thus their biliary specification to cholangiocytes. Alternatively, during hepatocyte regeneration, macrophage engulfment of hepatocyte debris induced Wnt3a expression. This resulted in canonical Wnt signaling in nearby HPCs, thus promoting their specification to hepatocytes [49].

## 6. Cellular Cross-Talk between HPC and HSC in Fibrogenesis

Studies of NAFLD, both in rodent models and human beings, have confirmed that HPCs are activated when oxidative stress inhibits the regenerative capacity of more mature hepatocytes supporting the concept that HPC expansion is a component of the liver’s adaptive response to oxidative stress [62,63]. Recent evidence suggested that resident stem/progenitor cell pool participates in the repair of liver damage either through the replacement of dead cells or by driving fundamental repair processes, including fibrosis and angiogenesis [38,64,65].

In this context, HPC activation and the expansion of ductular reaction (DR: Figure 1) have been independently correlated with progressive fibrosis in adult and pediatric NASH and in HCV related cirrhosis [38,39]. In adult human NASH, it has been proven that DR is strongly and independently correlated with progressive portal fibrosis raising the possibility of a second periportal pathway for fibrogenesis in NASH that is independent of the deposition of zone 3 subsinusoidal collagen by stellate cells. In nonalcoholic steatohepatitis (NASH), portal fibrosis is a recognized key feature associated with progression of the disease and represents the predominant form of fibrosis in some cases of pediatric nonalcoholic fatty liver disease (NAFLD) [30,34,36,39]. Recent results in pediatric subjects confirmed data on adult samples [38]. In these patients, the expansion of HPCs compartment is independently, at the multivariate logistic regression analysis, correlated with the degree of fibrosis indicating that also in pediatric NASH, DR is a main driver of fibrosis. Interestingly, HPC activation is correlated with hepatocyte apoptosis and cell cycle arrest induced by long lasting oxidative stress [38]. Accordingly, in NASH livers but not simple steatosis, a population of intermediate hepatocytes appeared. The presence of an intermediate hepatocyte (IH) pool was an additional novel finding of this study. IHs are intermediate cells between progenitors and mature hepatocytes and are characterized by intermediate size and faint cytokeratin-7 (CK7) immunoreactivity [41]. The appearance of IHs is a common aspect in other acute and chronic liver diseases and represents a sign of HPC differentiation towards hepatocyte lineage [41]. In pediatric NAFLD, the number of IHs was directly associated with the number of HPCs as well as the presence of hepatocyte ballooning and NAFLD activity score (NAS). These features suggest that, in NASH, the stimulation of the HPC compartment was associated with the production of IHs indicating that the differentiation of HPCs toward hepatocytes takes place [38].

Taken together, these observations indicated that, in the progression of NAFLD, the prolonged hepatocyte apoptosis and cell cycle arrest induced by oxidative stress can trigger the proliferation and activation of HPCs [38]. This determined the appearance and expansion of reactive ductules which activate fibrogenesis and angiogenesis processes (niche expansion) leading to periportal fibrosis [38,39].

In this context, DR could modulate hepatic fibrogenesis during liver injury through several mechanisms: (i) cells of DR are able to produce agents that are chemotactic for inflammatory cells and may activate HSCs [66,67]; (ii) cells of DR might undergo to epithelial-mesenchymal transition contributing to the portal myofibroblast pool [66,68].

The molecular cross-talk between the ductular reaction and activated stellate cells and myofibroblasts has been shown both in experimental models and in humans [69]. In general, reactive ductules have been demonstrated as a source of factors (such as Platelet-Derived Growth Factor, TGF-β, and Sonic Hedgehog) which are able to activate HSCs. In an experimental model, newly formed bile ductules were found to express MCP-1 and PDGF-β chain [65,70,71], capable of recruiting and activating HSCs to produce collagen (Figure 3) [72].

In several human liver diseases, proliferating ductular reaction was shown to express similar cytokines, including TGF-β1 and PDGF [73]. In submassive hepatic necrosis, proliferating HPCs increased their expression of profibrogenic factors and intimately localize with activated stellate cells or myofibroblasts [74].

In addition, new evidences indicate the possibility of epithelial to mesenchymal cell transition of cells participating in ductular reaction, suggesting that a portion of the myofibroblast pool may be derived from the phenotypic transformation of proliferating cholangiocytes and HPCs [66,68].

## 7. Role of Kupffer Cells and Macrophages and Their Cross-Talk with HPC and HSC in NAFLD

Macrophages play an essential role during the disease process of NAFLD by communicating inflammatory signals by scavenging modified lipids. Clinical findings and experimental data have demonstrated that activation of Kupffer cells (KCs) is a central event in the initiation of liver injury [75,76]. KCs, the liver resident macrophage pool, can accumulate large amounts of lipids, transform into foam cells and drive progression towards steatohepatitis (Figure 1). Recently, the process of macrophage polarization has been a subject of interest as macrophage subsets have been demonstrated to display some degree of plasticity and heterogeneity [77,78].

Two distinct modes of macrophage activation were proposed to differentiate between inflammatory M1 and anti-inflammatory M2 macrophages [79]. M1- and M2-macrophage subsets are generated in different inflammatory conditions. *In vitro*, the treatment of un-polarized macrophage with interferon (IFN)-γ and tumor necrosis factor (TNF)-α results in the generation of M1-macrophages that strongly produce pro-inflammatory cytokines (such as IL-1β, IL-6, IL- 8, IL-12, and TNF-α). M1-macrophage exerts definitive pro-inflammatory roles and M1-derived cytokines may play a role in further activating portal myofibroblasts and hepatic stellate cells. On the other side, macrophage can be polarized toward alternative activation phenotypes (M2) by IL-1β, IL-4, IL-13, and IL-10 cytokines. In general, M2-macrophages have been described as wound-healing macrophages, based on their ability to promote wound healing through matrix remodeling and the recruitment of fibroblasts [80]. M2-secreted cytokines may support the generation of anti-inflammatory Th2 cells, favoring alternative inflammation. Finally, M2-macrophages seem to be unable to efficiently phagocyte oxLDL but can secrete a variety of MMPs (MMP2, MMP9, MMP12, MMP13, MMP14) suggesting that M2-macrophages may promote the clearance of apoptotic cells.

M1-polarized macrophages play a key role in a variety of chronic inflammatory diseases, such as atherosclerosis [2], inflammatory bowel disease [81], or insulin resistance associated with obesity [82]. The exacerbated release of M1 Kupffer cell derived mediators contributes to the pathogenesis of several liver lesions, namely hepatocyte steatosis and apoptosis, inflammatory cell recruitment, and activation of fibrogenesis [76,83].

Moreover, recent evidences indicated a cross-talk between liver macrophage/Kupffer cell and HPCs in the regulation of HPC activation [83] and fate choice [49]. Liver macrophages are a source of Wnt. Ablation of macrophages during hepatocyte regeneration removed the stimulus for HPCs to become hepatocytes; instead, they differentiated into cholangiocytes and formed biliary structures. Notably, phagocytosis of the hepatocyte debris promoted profound Wnt upregulation in macrophages, providing a critical link between hepatocyte death and HPC fate that enables co-ordinated and appropriate tissue renewal [49].

A recent paper by Wan J. and colleagues indicated that favoring M2 KC polarization might protect against fatty liver disease [76]. Individuals with limited liver lesions displayed higher hepatic M2 gene expression and negligible hepatocyte apoptosis, as compared to patients with more severe lesions. Moreover, in mice models of fatty liver injury, genetic or pharmacological interventions favoring preponderant M2 KC polarization were associated with impaired M1 response and limited liver injury and a positive relationship between M2 KC polarization and M1 macrophage apoptosis [76].

Some emerging concepts indicate that the widely used M1/M2 macrophages classification does not address the more complex *in vivo* macrophage heterogeneity. In their recent work, Ramachandran P. *et al*. used Ly-6C expression to identify a macrophage subset responsible for the resolution of liver fibrosis (restorative macrophage) [84]. In particular, the analysis of Ly-6C expression identified two clearly distinct hepatic recruited macrophage populations: Ly-6C^high^ and Ly-6C^low^. Dynamic changes in these macrophage populations were seen during fibrogenesis and resolution [84].

Although Ly-6C^low^ restorative macrophages show increased expression of some M2 genes, they also down-regulate other typical M2 genes and, simultaneously, up-regulate some traditional M1 genes [84]. Therefore, these hepatic macrophage subpopulations do not fit into the M1/M2 classification and represent newly identified macrophage phenotypes, highlighting the limitations of this classification in an *in vivo* setting [84].

Since this study has been carried on murine model of hepatic fibrosis, a future goal is represented by the identification of analogous populations in cirrhotic human liver. This analysis is indispensable prior to extending these findings to human pathologies.

## 8. Adipokines as a New Tool in HPC and HSC Cross-Talk in NAFLD

The term “adipokines” (adipose tissue cytokines) comprises polypeptide factors which are expressed significantly, although not exclusively, by adipose tissue in a regulated manner [85]. Recently, hepatic progenitor cells have been indicated as a source of adiponectin and resistin in the course of NAFLD [38].

Parallel with their expansion in NASH, HPCs down-regulated their expression of adiponectin. The inverse correlation between adiponectin and NASH progression is in agreement with the current understanding of this adipokine [86]. In fact, adiponectin has anti-inflammatory and anti-fibrogenic properties and, in steatotic liver, has been showed to ameliorate necroinflammation and steatosis when administered in experimental NASH [85,86]. On the other hand, HPCs up-regulated their expression of resistin in correlation with progression towards NASH and fibrosis [87]. Several lines of evidence link the biology of resistin with hepatic inflammation, fibrogenesis and macrophage polarization. In rats, resistin administration significantly worsens inflammation after lipopolysaccharide injection [88], and activated human HSCs respond to resistin with increased expression of proinflammatory chemokines and nuclear factor-kappa B activation [86,88]. Hepatic resistin expression increases in alcoholic steatohepatitis and NASH and is correlated with inflammatory cell infiltration. Resistin has been particularly associated with macrophage recruitment within the liver; this relationship could be related to the release of MCP-1 which contributes to macrophage infiltration.

Indeed, adiponectin and resistin could represent a new key tool in the cellular cross-talk among HPCs, HSCs and liver macrophages. Moreover, modification of hepatic adipokines and GLP-1 production by HPCs and/or hepatocytes could have a role in the progression of insulin resistance (IR) and NASH [89,90]. IR is an important pathogenic factor in the development and progression of nonalcoholic fatty liver disease. The metabolism of lipid in the hepatocytes is controlled by hormones such as insulin and by locally generated factors, and represents the result of complex interactions among multiple cell types located in different tissues [90]. Insulin activates the insulin receptor tyrosine kinase, which subsequently phosphorylates IRS1 and 2 [90]. Through a set of intermediary steps, this leads to activation of Akt2. Akt2 can promote glycogen synthesis, suppress gluconeogenesis, and activate *de novo* lipogenesis [90].

This central signaling pathway could be altered by several mechanisms leading to hepatic insulin resistance. Fatty infiltration of the liver is closely linked to IR. Insulin is a potent inhibitor of hepatic endogenous glucose production [90]. Lipid-induced insulin resistance implicates the diacylglycerol-mediated activation of protein kinase C (PKC)-ɛ, and subsequent impairment of insulin signaling increased sequestration of Akt2. Impaired Akt2 activation increases expression of key gluconeogenesis enzymes [90]. Impaired Akt2 activity also decreases insulin-mediated glycogen synthesis. Several intracellular inflammatory pathways could be also implicated in hepatic insulin resistance such as the activation of IKK by TLR4 and the activation of JNK1 by TNF-α [90]. Moreover, genetic and molecular studies support a critical role for PTEN in hepatic insulin sensitivity and the development of steatosis, steatohepatitis and fibrosis [91].

Finally adipokines have a key role in IR [85,89]. In fact, adiponectin is able to suppress hepatic glucose production, to improve insulin signaling, and exerts insulin-sensitizing effects in the liver. By contrast, resistin can increase endogenous glucose production by the liver, induction of insulin resistance and stimulation of proinflammatory cytokines. Finally, GLP1 has insulin-independent effects on glucose disposal in extra-pancreatic tissues, including the liver. In hepatocytes, GLP1 activates glycogen synthesis and has been implicated in the regulation of glucose homeostasis and insulin resistance in animal models of NAFLD [85,89].

## 9. NAFLD and Atherosclerosis: Possible Molecular Mechanisms

Several clinical and experimental evidences underscore that atherosclerosis and NAFLD share multiple cellular and molecular pathogenetic mechanisms [92]. In this context, the liver is both the target of and a contributor to systemic inflammatory changes. Several studies have shown that a number of the genes involved in fatty acid metabolism, lipolysis, monocyte and macrophage recruitment, coagulation, and inflammation are overexpressed in patients with nonalcoholic fatty liver disease [93]. Altered transcriptional regulation of pro-atherogenic genes occurs in the liver of patients suffering from NASH and it is associated with the activation of molecular events that may also be responsible for the local production of mediators or modifiers of circulatory homeostasis [5]. In particular, NASH, but not simple steatosis, is associated with the regulation of genes in the liver which are associated with atherosclerotic risk and, as such, may contribute to the pro-atherogenic state [5]: circulating levels of several inflammatory markers (*C*-reactive protein, interleukin-6, monocyte chemotactic protein 1, and TNF-α), procoagulant factors (plasminogen activator inhibitor 1, fibrinogen, and factor VII), and oxidative stress markers are highest in patients with NASH, intermediate in those with simple steatosis, and lowest in control subjects without steatosis [92,93].

These observations strongly suggest that non-alcoholic steatohepatitis can contribute to a more atherogenic risk profile over and above the contribution of visceral adiposity [94]. In this light, liver is both the target of systemic abnormalities and a source of pro-atherogenic molecules that amplify the arterial damage, thus resulting in the accelerated atherogenesis observed in NAFLD patients [92].

## 10. Conclusions

The pathogenesis of NAFLD is described by the “two-hit” hypothesis first proposed in 1998 [95]. The “first hit” (*i.e.*, fat accumulation) sensitizes the liver to the injurious effects of one or more additional factors, while the “second hit” leads to the development of steatohepatitis and fibrosis. The “second hit” could be represented by a variety of factors and determinates the development of inflammation (NASH) and fibrosis. However, this variety of factors (second hit) could act on several cell types through intra- and inter-cellular cross-talks which remain mostly unknown. The characterization of intricate interactions between resident and recruited cells represents a key aspect to understanding the mechanisms underlying damage progression towards NASH and cirrhosis. The growing consensus is that the cross-talk between hepatocytes, hepatic stellate cells and macrophages in NAFLD plays a main role in the derangement of lipid homeostasis, insulin resistance, danger recognition, immune tolerance response, and pericentral fibrogenesis. On the other hand, the activation of hepatic progenitor cell niche by hepatocyte apoptosis and cell-cycle arrest has a central role in the stimulation of portal myofibroblasts determining the development of periportal fibrosis.
